# Decoding electroencephalographic responses to visual stimuli compatible with electrical stimulation

**DOI:** 10.1063/5.0195680

**Published:** 2024-06-12

**Authors:** Simone Romeni, Laura Toni, Fiorenzo Artoni, Silvestro Micera

**Affiliations:** 1 Modular Implantable Neurotechnologies (MINE) Laboratory, Università Vita-Salute San Raffaele & Scuola Superiore Sant'Anna, Milan, Italy; 2 Bertarelli Foundation Chair in Translational Neural Engineering, Center for Neuroprosthetics and Institute of Bioengineering, Ecole Polytechnique Federale de Lausanne, Lausanne, Switzerland; 3 The Biorobotics Institute and Department of Excellence in Robotics and AI, Scuola Superiore Sant'Anna, Pontedera, Italy; 4 Department of Clinical Neurosciences, Faculty of Medicine, University of Geneva, Geneva, Switzerland

## Abstract

Electrical stimulation of the visual nervous system could improve the quality of life of patients affected by acquired blindness by restoring some visual sensations, but requires careful optimization of stimulation parameters to produce useful perceptions. Neural correlates of elicited perceptions could be used for fast automatic optimization, with electroencephalography as a natural choice as it can be acquired non-invasively. Nonetheless, its low signal-to-noise ratio may hinder discrimination of similar visual patterns, preventing its use in the optimization of electrical stimulation. Our work investigates for the first time the discriminability of the electroencephalographic responses to visual stimuli compatible with electrical stimulation, employing a newly acquired dataset whose stimuli encompass the concurrent variation of several features, while neuroscience research tends to study the neural correlates of single visual features. We then performed above-chance single-trial decoding of multiple features of our newly crafted visual stimuli using relatively simple machine learning algorithms. A decoding scheme employing the information from multiple stimulus presentations was implemented, substantially improving our decoding performance, suggesting that such methods should be used systematically in future applications. The significance of the present work relies in the determination of which visual features can be decoded from electroencephalographic responses to electrical stimulation-compatible stimuli and at which granularity they can be discriminated. Our methods pave the way to using electroencephalographic correlates to optimize electrical stimulation parameters, thus increasing the effectiveness of current visual neuroprostheses.

## INTRODUCTION

Electrical stimulation (ES) can elicit visual sensations in a large number of patients affected by acquired blindness.^1,2^ Stimulation can be delivered via retinal^3,4^ or optic nerve^5,6^ prostheses for retinal degeneration or cortical prostheses^7^ for optic neuropathies or stroke-induced blindness. In most visual prostheses, a camera captures a stream of visual scenes, which are first processed through computer vision algorithms^8–12^ and then converted to stimulation patterns through an *a priori* model of the visual sensations (phosphenes) produced by stimulation. In retinal stimulation, it is customary to model phosphenes as round and to build stimulation protocols from “pixelizations” of the target visual scenes,^13,14^ resulting in suboptimal stimulation protocols as this assumption is only approximately true.^15^ The “pixelization” approach is even more suboptimal in the case of optic nerve stimulation because of the very elongated phosphenes generated in this case.^16^

In Ref. 17, we employed neural network models inspired DiCarlo and colleagues' work^18,19^ to show *in silico* that the ES of the optic nerve may be optimized by the cortical activation elicited by stimulation as a feedback signal. A similar approach has been proposed *in vivo* in mice,^20^ with both stimulation and recordings at the same cortical area. Such “end-to-end” optimization strategies evaluate the performance of different candidate stimulation protocols implicitly taking into account the limits of current interfaces, while in other encoding techniques the construction of stimulation targets does not take them into account. Moreover, such optimization process does not require knowledge of all the anatomical and functional details necessary to build a detailed biophysical model like the ones available for animal models, which still require very invasive or destructive imaging techniques.^21^

Here, we investigate whether electroencephalography (EEG) can be used as a feedback cortical signal to optimize ES protocols, allowing stimulation protocol optimization without resorting to invasive procedures except for the neuroprosthesis implantation. EEG has been used in the past to characterize the cortical response to electrical stimulation,^22–25^ but no framework has been formulated for automatic optimization based on EEG to the best of our knowledge. Even though EEG is an ideal candidate in terms of non-invasiveness, it is characterized by poor signal-to-noise ratio (SNR) and spatial resolution,^26^ which could prevent the discrimination of structural features of visual stimuli. In addition, in our case, we need to consider stimuli compatible with the perceptions elicited by ES (phosphenes), consisting of solid bright blobs on a uniform background. Increasing the stimulation amplitude of a given electrode contact increases the size of the solid blob and thus the average luminance of the stimulus (as it recruits more cells without substantially altering their firing rates^16,27^). This makes our ideal visual stimuli quite different from those employed in neuroscientific settings, where investigations have focused either on the semantic valence of “complex” images, representing, e.g., human faces, animals, and everyday-life objects,^28–31^ or on the response to structural variations and in different subject cohorts of “simple” stimuli like distributed gratings^32–36^ or checkerboards.^37–39^ In general, the different visual stimuli in such datasets vary the value of a single feature at a time, e.g., the size of isoluminant stimuli. This implies that the results obtained by existing studies may not be immediately translatable to our scenario and motivated us to acquire a new dataset of EEG correlates, characterizing the response to such phosphene-like visual stimuli. We then proceeded to investigate whether the distributions of the EEG correlates with several stimulus features of our visual stimuli were statistically distinguishable and to evaluate whether a classification algorithm could reliably assign an EEG response to a given feature class.

**FIG. 1.**
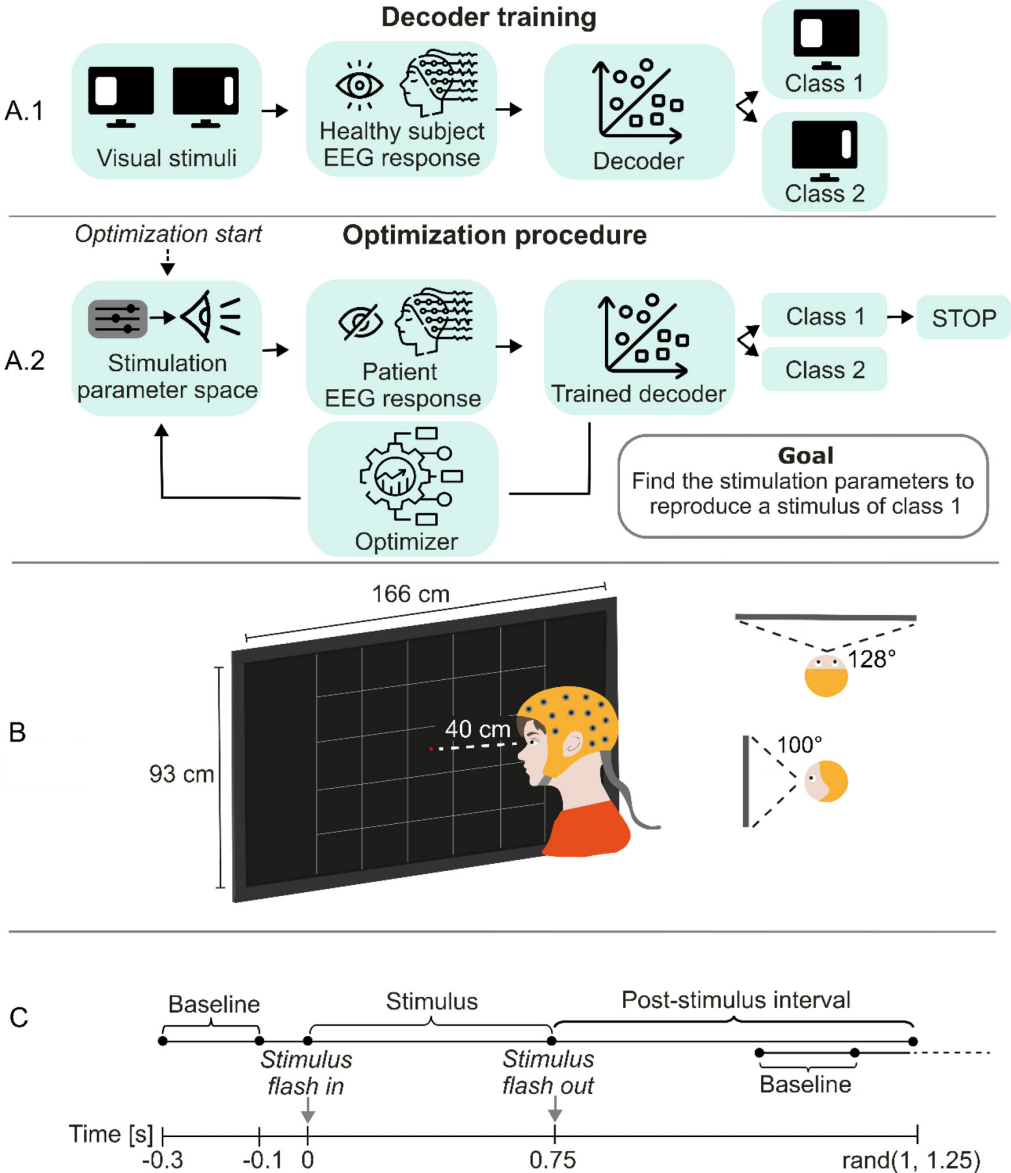
Experimental setup. (a) Workflow for the use of an EEG decoder of visual features in the optimization of electrical stimulation for vision restoration. (b) Positioning of the subjects with respect to the screen, screen dimensions, and covered portion of the visual field. Stimuli were flashed on a region of the screen, divided into 25 equally sized squares. Participants were asked to fixate the center of the screen where a red dot was presented. (c) Representation of the visual stimulation sequence, consisting of baseline, stimulus, and random post-stimulus intervals.

Such EEG response classifiers could be used to “evolve” ES protocols until the elicited response is assigned to a given target class as illustrated in Fig. 1(a). The use of an EEG decoder allows optimization of stimulation parameters relying on a quantitative marker and may substantially speed up the optimization process, as also discussed in Ref. 17. While such evolution should ideally happen on the basis of the decoding of single EEG trials, it is possible that the information from multiple trials may be required because of the very low SNR of EEG signals.^26^ Multi-trial decoding can be performed by simple concatenation of several trials along the time- or the channel-dimension or by training and testing the decoder on event related potentials (ERPs).^40^ The former approach leads to results dependent upon trial ordering unless complex training schemes are employed, while the latter approach requires a very large dataset, so that an acceptable number of samples remains after trial averaging. Here, instead, we aggregated the class probabilities for the single trials.^40,41^ We then characterized the improvement in decoding accuracy produced by using several trials at a time with respect to a single one.

## RESULTS

In the present work, we analyzed data from ten healthy subjects positioned in front of a screen covering most of their visual field [Fig. 1(b)]. We flashed different rectangular bright stimuli on a black background, for a total of 50 repetitions of each one of 60 different stimuli (see supplementary material Fig. 1). Each visual stimulus was flashed in and kept for 750 ms, then flashed off. The next stimulus was flashed in after an inter-stimulus time randomly chosen in the range of (1000 and 1250) ms [Fig. 1(c)]. In order to assess the possibility to discriminate several features of a stimulus, we defined a set of decoding tasks, respectively, to decode information about the location of the presented stimulus, its size, or both. To do this, we formed classes from different sets of the 60 original stimuli, and we determined ten decoding tasks where it was necessary to discriminate whether a stimulus belonged to one of several mutually exclusive classes. The set of stimulus classes and decoding tasks is detailed in supplementary material Fig. 2.

**FIG. 2.**
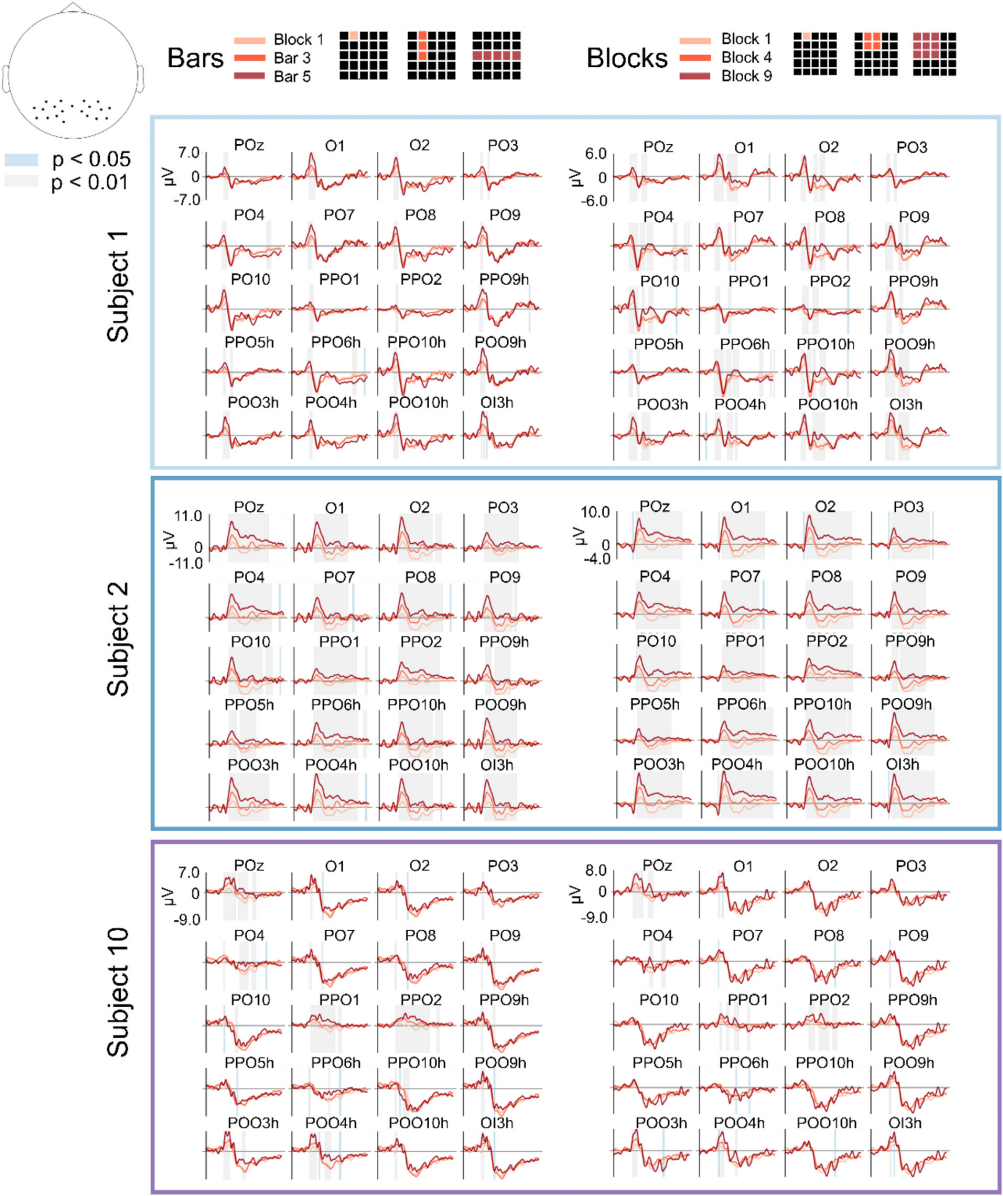
ERPs for luminosity-related classes. ERPs (0–0.75 s) for luminosity-related classes (1-block vs 3-bars vs 5-bars, and 1-block vs 4-blocks vs 9-blocks) for three subjects in the occipital and parietal channels. Chosen subjects (subjects 1, 2, and 10) are those that had best, worst, and average classification scores. Different colors correspond to different stimuli, while colored bands correspond to time intervals where the single-trial responses for the different classes are significantly different, with the band color representing the level of significance. The location of the shown channels is displayed in the topographic map in the top left.

**FIG. 3.**
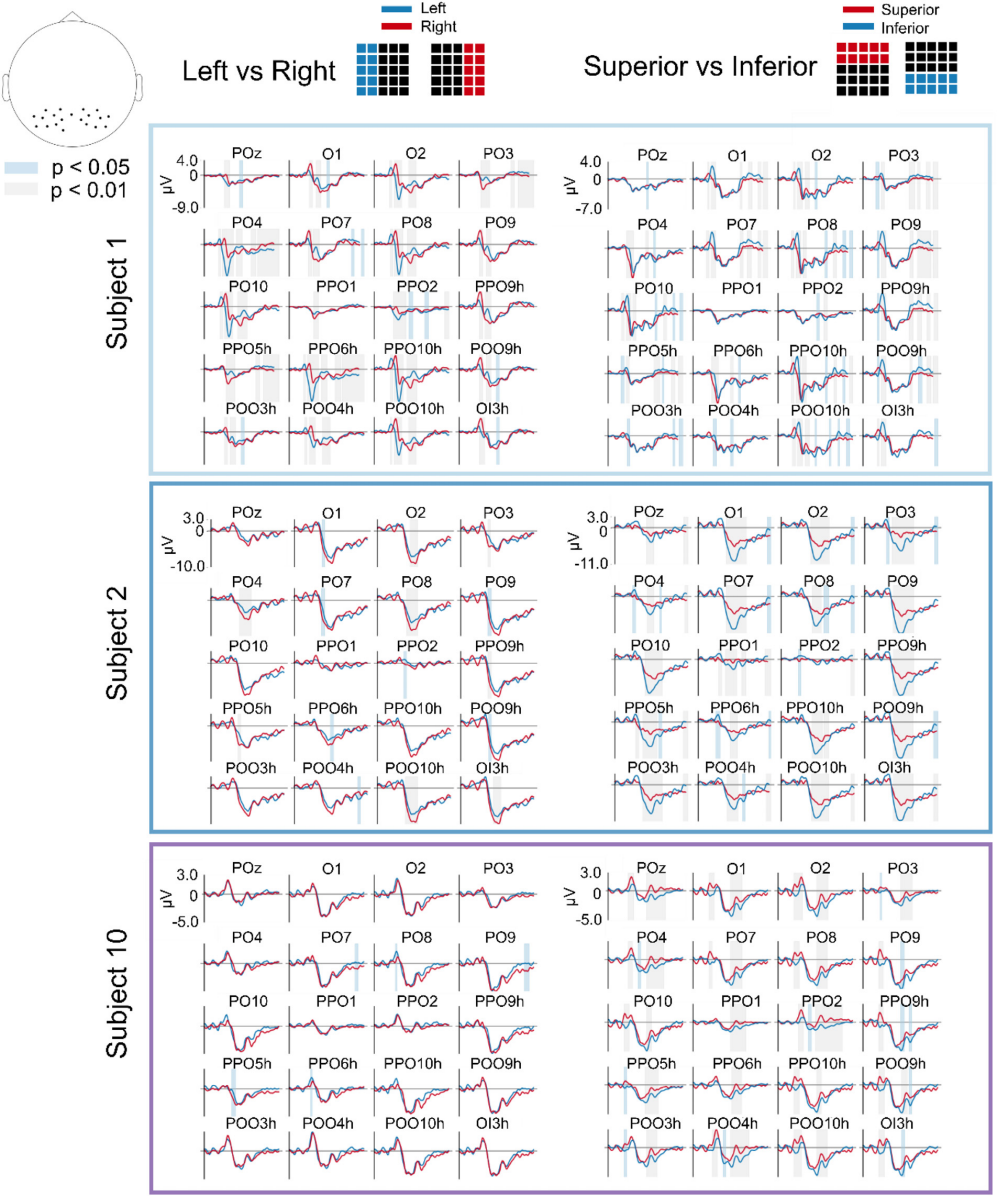
ERPs potentials for location-related classes. ERPs (0–0.75 s) for location-related classes (left vs right, and superior vs inferior) for three subjects in the occipital and parietal channels. Chosen subjects (subjects 1, 2, and 10) are those that had best, worst, and average classification scores. Different colors correspond to different stimuli, while colored bands correspond to time intervals where the single-trial responses for the different classes are significantly different, with the band color representing the level of significance (see legend). The location of the shown channels is displayed in the topographic map at the top left corner.

Figure 2 shows the ERPs in the occipital channels for the bars and blocks tuning sets, to give an example of modulations related to the size of the stimuli and thus to their luminosity. In general, larger visual stimuli produce a larger positive and a smaller negative deviation in the ERP with respect to baseline. This means that scaling the size of a given visual stimulus does not result in a scaled copy of the EEG response as it could be expected. The dependency of the ERP on the stimulus size is similar across different subjects, even considering inter-subject variations in the ratios between the peak amplitudes for different stimulus sizes. Instead, Fig. 3 displays the ERPs in the occipital channels for the coarse left vs right and superior vs inferior tuning sets, to give an example of modulations related to the location of the stimuli, irrespective of their size. The inter-subject differences in the ERPs corresponding to stimulus in different locations of the visual field are more marked. For example, stimuli in the left part of the visual field produced a larger negative deflection in the right part of the scalp, and stimuli in the lower part of the visual field produced larger negative deflections in the ERPs. In both cases, the time intervals where the responses for the different classes were significantly different are shown. For most tasks and subjects, several regions reached statistical discriminability (p < 0.05) among the different classes.

**FIG. 4.**
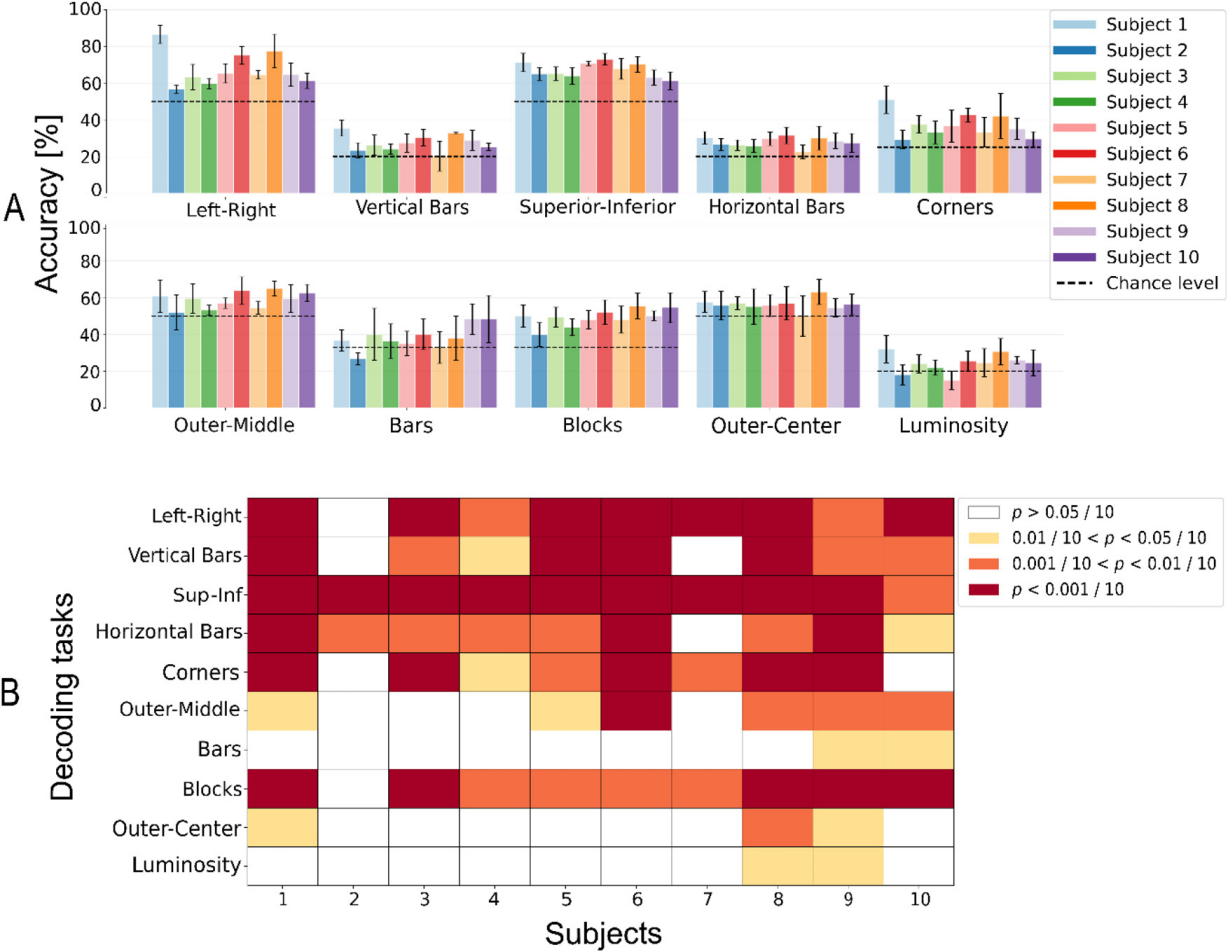
Single-trial decoding. (a) Classification accuracy for all subjects and decoding tasks, evaluated as the mean of a tenfold cross-validation or ten resamplings. Error bars represent the standard deviation over the ten folds and resamplings. (b) P-values obtained from the Wilcoxon test for all macro-classes and all subjects. The analysis tests if the median accuracy obtained with single-trial decoding is greater than that obtained with a random decoder. Cells have been highlighted in red if p < 0.0001, orange if p < 0.001, yellow if p < 0.005, and white if p > 0.005.

In Fig. 4(a), subject-wise accuracies for the tenfold cross validation folds or Monte Carlo resamples are shown with the corresponding chance level. As can be seen from Fig. 4(b), the decoding accuracy is significantly above chance both in the location decoding tasks and in the blocks decoding tasks. When accuracy is not significantly higher than chance for a task and subject, it tends to be so for more than one subject, suggesting an intrinsic difficulty of the decoding task, rather than a low signal quality from the specific subject. The decoding performance is variable across subjects, confirming an important inter-subject variability. As expected, we noticed that our decoding algorithm had worst performance for tasks that included many classes whose stimuli differ only slightly (e.g., blocks and bars, where the difference between bar3, block4, and bar5 is only one block), or when concurrent increases in luminosity and eccentricity of the stimulus were present. In the latter case, higher luminosity would tend to produce high ERP responses, while higher eccentricity would lead to decreased ERP amplitudes, possibly leading to a compensation of the two effects (out vs center decoding task).

Decoding scores for three subjects with and without ICA removal of ocular activity showed no substantial differences (supplementary material Fig. 3). In supplementary material Fig. 4, we show features' importance for the RBF SVM decoding for left vs right and superior vs inferior portions in subject 1. The complete map of features' importance and averaging across time to get a spatial map and across channels to get a temporal map of importance are displayed.

**FIG. 5.**
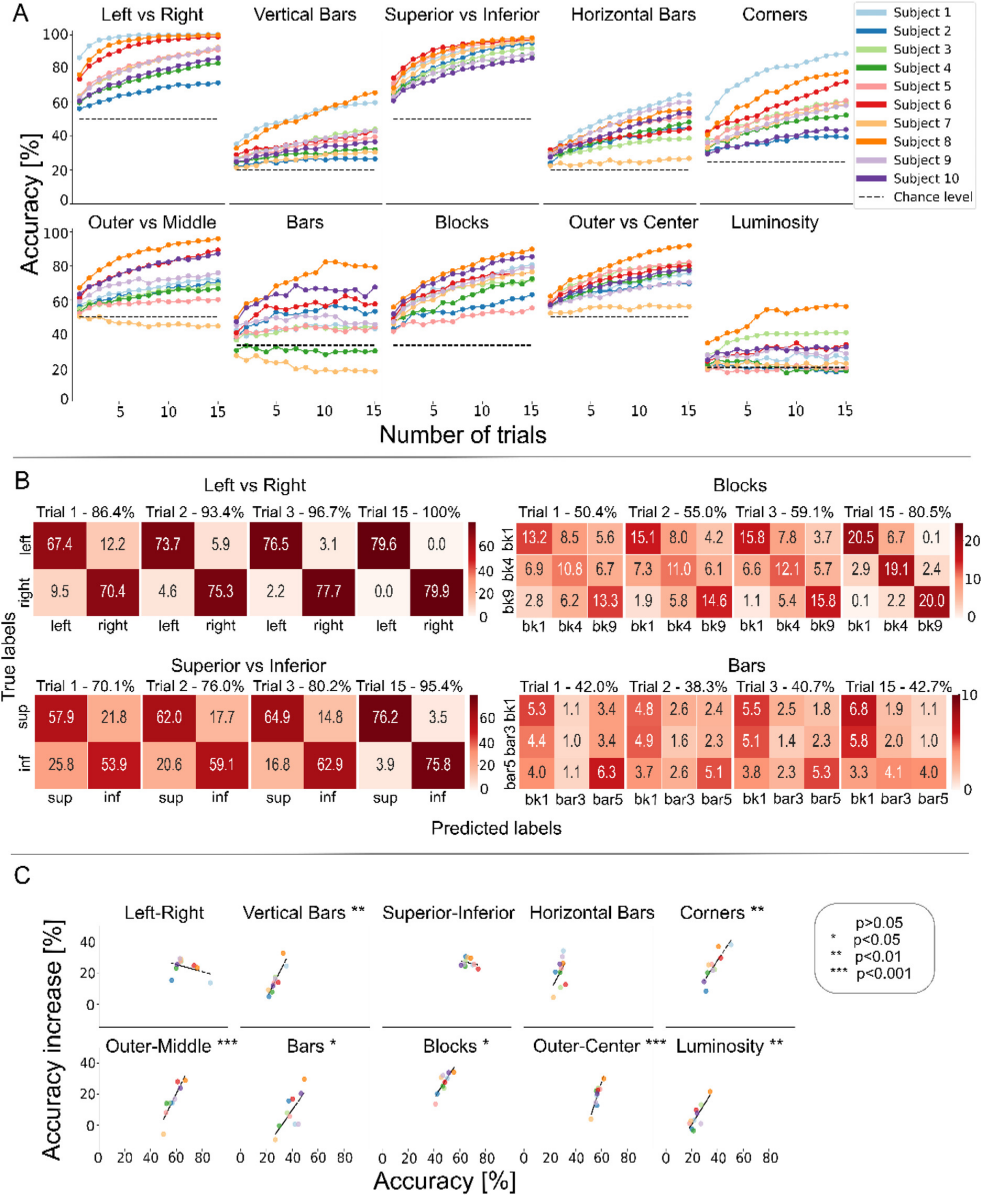
Multi-trial decoding results. (a) Subject-wise accuracy curves obtained by varying the number of trials used in the multi-trial inference. (b) Dependency of the variation of accuracy from single-trial to 15-trial (multi-trial) inference upon the single-trial variation. Positive values account for an increase in accuracy between single- and multi-trial inference. The black line represents the least squares fit of the points in each subplot, and the levels of significance associated with the line slopes are reported in the subplot titles. (c) Average confusion matrices for subject 1, single-, 2-, 3-, and 15-trial inference. Averages are computed across the tenfold cross validation/10 Monte Carlo resamplings. In the subplot titles, we report the average accuracy across folds.

In Fig. 5(a), it is possible to observe how the performance of multi-trial decoding varies when an increasing number of trials is employed in the decoding. Multi-trial decoding can lead to important increases in accuracy, even if different subjects lead to different performance increases. Interestingly, in some cases multi-trial decoding led to a decrease in accuracy. To investigate such phenomenon, the performance for single- and multi-trials for each cross-validation fold or Monte Carlo resampling is shown in supplementary material Fig. 5, together with the confidence interval of the random decoder for the given task and subject. We then investigated whether there is a dependency of the accuracy variation led by multi-trial decoding upon the initial accuracy and show the results of this analysis in Fig. 5(b). When such dependency was statistically significant, we found a positive correlation between the two variables, meaning that multi-trial decoding produced higher performance increases with respect to single-trial decoding when the initial performance of the single-trial was higher.

## DISCUSSION

In this work, we defined a novel set of visual stimuli compatible with ES of the visual system and showed that the EEG ERPs corresponding to different stimulus classes are statistically distinguishable. We then performed single-trial decoding using traditional ML, obtaining above chance-level accuracies, and improved them with a multi-trial decoding strategy.

The ability to decode visual stimuli from EEG activity is a premise for the optimization of the ES using such technique as feedback. In fact, it can allow determination of whether the applied electrical stimulation produces a sensation with specific target features without the need to rely on subjective feedback collected via questionnaires. The optimization process exploits a set of decoders trained on healthy subjects, where the association between visual perception and EEG correlate is known, as it corresponds to the visual stimulus generating the EEG response. The optimization loop would thus consist in choosing a target perception, applying a stimulation protocol on the patient, characterizing the features of the elicited perception passing the EEG evoked activity through the above-mentioned decoders (establishing whether the evoked perception occupies the left or right, superior or inferior visual field), and proposing the next candidate stimulation to minimize the discrepancy between the features of the evoked and target perceptions.

In our analyses, preprocessing was kept to a minimum, i.e., to bad channel rejection and frequency filtering. This is coherent with our final goal of using such analyses to optimize visual stimulation in real-time. Indeed, bad channel rejection can be performed at the beginning of acquisition. During the optimization experiments, after each candidate stimulus is administered, the corresponding EEG epoch is filtered (which can be done in real-time) and passed through a set of decoders establishing its similarity to a given target natural stimulus so that the next candidate stimulus is determined. Instead, the application of other state-of-the-art methods to improve signal quality like, e.g., ICA can be time intensive and normally requires expert input (e.g., manually selecting artefactual independent components), which would add a substantial amount of time between the presentation of different candidate stimuli, reducing the amount of explorable candidates during an experimental session. Additionally, recent works report that the use of such techniques often leads to marginal or no improvement in the target application,^42^ thus not justifying a drastic reduction in the throughput of our optimization routine.

The presented ERPs do not show the typical peaks studied in the literature related to visual responses like N70 and P100. It is interesting to remark that even though not visible in the ERPs, the information present at such latencies is exploited by the employed RBF SVMs, as we observed peaks of feature importance in those locations, particularly evident in occipital and parietal areas, corresponding to visual sensory areas. Thus, the peaks could be covered by the surrounding neural activity and not be visible through averaging but they may be highlighted by the nonlinear processing performed by our kernel machine. The very small amplitude of the components linked to lower sensory cortical areas may be linked to the fact that they are generally observed in correspondence to variations in stimulus spatial frequency and orientation.^43^ Additionally, it is known that stimuli of large size trigger surround suppression in lower visual areas because of the tendency of neurons to suppress the activation of other neurons with neighboring receptive fields.^44–46^ Responses with latencies similar to ours have been found in healthy controls under visual stimulation in Ref. 47.

Typically, EEG decoding is performed either by extracting features defined in the literature and passing them to traditional machine learning or by passing the whole spatiotemporal signal to deep learning (DL) architectures. In our case, neither strategy was feasible. The lack of knowledge about the present class of stimuli prevented us from extracting meaningful features, while the small set of collected data prevented the use of DL techniques. We thus chose a “hybrid” approach, feeding each spatiotemporal data point (as an independent feature) to SVMs, which are known to display low sensitivity to irrelevant features^48^ and are one of the most widely used state-of-the art techniques for EEG decoding.^49–51^ While several automatic and semiautomatic denoising techniques that could (at least in principle) improve our decoding performance exist, their contribution is debatable in the general case.^42^

Another aspect contributing to the choice of a relatively simple ML algorithm is the speed of prediction, which would have been substantially higher with DL or even recurrent architectures. Decreasing the prediction speed decreases the number of stimuli that can be administered during an optimization run, which could lead to worse optimal stimuli than in the case of longer optimization runs with higher performance decoders. The interplay between decoder performance and prediction time and thus the ability to explore more candidate optima should be tackled in future developments of the present work.

Here, we gave a quantitative idea of what tasks lead to a successful decoding (corners, five vertical and horizontal lines) and which ones are too difficult given our simple decoding strategy (luminosity, out vs center). The two most difficult tasks were characterized by an elevated number of target classes (luminosity) or by the concurrent variation of stimulus size and location. The latter is particularly difficult as, in general, stimuli that occupy more peripheral portions of the visual field excite a lower number of photoreceptor and thus of neurons in higher areas because of the foveated nature of the human retina,^52,53^ which also happens in the case of smaller visual stimuli. Thus, we have a partial compensation of the two effects of moving peripherally and increasing the size of a stimulus, negatively affecting the decoder performance.

When we referred to the decoding of the stimulus size, what was actually decoded depended on a combination of size, luminosity, and luminance. We could have chosen stimuli where size was the only feature that varied, e.g., by using a black-and-white checkerboard pattern on a gray background, as in most studies in the neuroscience of vision field. Here, we did not apply a correction for stimulus luminosity as we were not interested in determining the neural correlates of given visual features in isolation, such as stimulus size and location. Instead, we strived to determine whether EEG could be a technique capable of discriminating visual stimuli compatible with ES and simple enough to be composed to build and study simplified visual scenes in the future. ES produces solid light blobs whose luminosity, size, and location are intertwined and related to the chosen stimulating contact in the implanted electrode and the stimulation protocol and amplitude.^16^ Crafting a set of visual stimuli in which single stimulus features are maximally decoupled and distinguishable could still be of translational interest as it could allow the implementation of better feature extractors preceding decoding. Other than increasing the resulting decoding accuracy, this could reduce the number of trials required to attain a given performance, speeding up the process of stimulation optimization and thus decreasing the burden for the target subject.

Multi-trial decoding generally led to an increase in performance, except in cases in which the single-trial performance was not significantly higher than chance. In those cases, we did not observe systematic worsening of the performances but rather unpredictable behavior. All of this is reasonable, since the presented multi-trial approach employs calibrated classification probabilities. When the probability of correct classification is not above chance, the classification probabilities are not informative and the outcome when merging is unpredictable. Moreover, the above observation serves as a rule of thumb for establishing when to employ multi-trial decoding: when single-trial performance is significantly above chance.

While the visual stimuli employed in this work are much simpler than the visual scenes that the healthy is confronted with in everyday life, they are not so far from what is possible through current visual neuroprostheses.^6,54,55^ In particular, existing visual neuroprostheses provide limited resolution, given by inter-electrode crosstalk and by the hardware complexity to accommodate high electrode counts in the retina or optic nerve because of anatomical reasons.^56^ To cope with such limited resolution, it is customary to heavily simplify the scenes coming from an external camera, focusing on reconstruction of the shapes and locations of a few salient objects and omitting most of the structural details.^57,58^

We remark that decoders trained on a cohort of healthy subjects may not be usable out of the box on patients because of the differences between the cortical correlates of electrical and visual stimulation and of the possible plastic modifications that occurred in patients after the insurgence of blindness.^59,60^ In both cases, extensive characterization of the neuroprosthetic device after implantation will be needed, mapping simple stimuli to the evoked sensations determined through patient feedback. Then, transfer learning in the form of fine-tuning of the decoders on patient data (correspondence between visual perception evoked through electrical stimulation and corresponding evoked EEG) and domain adaptation techniques (matching the distribution of responses from healthy subjects and target patient) could be used to facilitate generalization.

## METHODS

### Experimental setup

EEG signals were acquired from a 128-channel gel-based ANT Neuro cap using eego64™ software. We acquired data from ten healthy subjects (3 females), 23 ± 2 years old (average ± standard deviation). All participants had normal or corrected-to-normal vision and no history of neurological disorders. They were positioned in front of a 75 in. screen (166 × 93 cm^2^) at a distance of 40 cm, so that the screen covered a field of view of 100° horizontally and 128° vertically [Fig. 1(b)].

Each subject performed two experimental sessions lasting approximately one hour on different days. Each experimental session was divided into 30 sub-sessions consisting of the administration of 50 visual stimuli each. Subsequent sub-sessions were separated by a resting period controlled by the subject (on the order of a few minutes). Each visual stimulus was flashed in and kept for 750 ms, then flashed off. The next stimulus was flashed in after an inter-stimulus time randomly chosen in the range of (1000 and 1250) ms [Fig. 1(c)]. During each sub-session, subjects were asked to fixate a red dot in the center of the screen so that the administered visual stimuli spanned their whole visual field.

For each experimental session, we administered 25 repetitions of each one of 60 different visual stimuli, representing gray (luminosity equals 0.5) squares and rectangles with different sizes, locations, and orientations (vertical and horizontal) on a black background (luminosity equals 0). The complete set of visual stimuli is shown in supplementary material Fig. 1.

### Data preprocessing

For each session, we removed EEG channels with an impedance higher than 100 kΩ (5 channels on average). We acquired signals at a sampling frequency of 1 kHz. We bandpass filtered the continuous recordings from each session between 0.5 and 30 Hz to remove low frequency drifts, including bands up to beta. Specifically, we used the mne.Raw.filter function to create a one-pass, zero-phase, non-causal FIR filter designed using window method with Hamming window, with lower and upper cutoff frequencies (defined as half-amplitude cutoff in the middle of the transition band) 0.25 and 33.75 Hz, respectively. For each stimulus presentation, we considered the signal between –0.3 and 0.75 s, with respect to the stimulus onset. We will refer to the signal between 0 and 0.75 s as an epoch or trial. In symbols,

$$X_{j}^{nm}\left[t\right]=X_{j}^{m}\left[\left\lfloor \left(T_{n}-0.3\right)\cdot f_{s}\right\rfloor +t\right],\;\text{for}\;t=1,\;\ldots ,\;\left\lfloor 1.05\cdot f_{s}\right\rfloor \in N,$$

where $\left\lfloor \cdot \right\rfloor$ indicates the floor function, thus $\left\lfloor \tau \cdot f_{s}\right\rfloor$ refers to the sample number at time τ seconds. $T_{n}$ is the time of occurrence of the nth stimulus presentation, while j indicates the recording channel, and m the subject.

We performed baseline correction by subtracting from each epoch the average value of its baseline, taken in the interval (−0.3, −0.1) s,

$$X_{j}^{nm}[t]\leftarrow X_{j}^{nm}\left[t+\left\lfloor 0.3\cdot f_{s}\right\rfloor \right]-\frac{1}{\left\lfloor 0.2\cdot f_{s}\right\rfloor }\sum_{i=1}^{\left\lfloor 0.2\cdot f_{s}\right\rfloor }X_{j}^{nm}\left[i\right].$$

Then, we performed another step to reject bad channels: for each channel, we computed the average of the RMS value over all epochs, or

$$\mathrm{aRM}S_{j}^{m}=\frac{1}{N_{n}}\sum_{n=1}^{N_{n}}\left\{\sqrt{\frac{1}{N_{t}}\sum_{t=1}^{N_{t}}{\left(X_{j}^{nm}\left[t\right]\right)}^{2}}\right\}.$$

We removed from the dataset all channels with outlier average RMS (aRMS), namely, all the values of j so that

$$\mathrm{aRM}S_{j}^{m}>q\left({\left\{\mathrm{aRM}S_{j^{\prime }}^{m}\right\}}_{j^{\prime }},\;3\right)+1.5\cdot \mathrm{IQR}\left({\left\{\mathrm{aRM}S_{j^{\prime }}^{m}\right\}}_{j^{\prime }}\right),\;\mathrm{or}$$

$$\mathrm{aRM}S_{j}^{m}<q\left({\left\{\mathrm{aRM}S_{j^{\prime }}^{m}\right\}}_{j^{\prime }},\;1\right)-1.5\cdot \mathrm{IQR}\left({\left\{\mathrm{aRM}S_{j^{\prime }}^{m}\right\}}_{j^{\prime }}\right),$$

where $q\left(S,\;k\right)$ indicates the kth quartile of the set of values S, and IQR is the interquartile range,

$$\mathrm{IQR}\left(S\right)=q\left(S,3\right)-q\left(S,\;1\right).$$

### Stimulus classes

In order to assess the possibility to discriminate several features of a stimulus, we defined a set of decoding tasks, respectively, to decode information about the location of the presented stimulus, its size, or both. To do this, we formed classes from different sets of the 60 original stimuli, and we determined ten decoding tasks where it was necessary to discriminate whether a stimulus belonged to one of several mutually exclusive classes. The set of stimulus classes and decoding tasks is detailed in supplementary material Fig. 2. The decoding tasks “left vs right” and “superior vs inferior” discriminated stimuli belonging, respectively, to the left or right portion and superior or inferior portion of the screen grid and thus to the subject visual field. The tasks “vertical bars” and “horizontal bars” insert a finer granularity in the decoding of the vertical or horizontal location of a stimulus in the subject visual field, introducing five levels (two blocks left to the center, one block left to the center, center, one block right to the center, two blocks right to the center). The tasks “corners” and “outer vs middle” allow to further investigate the possibility of decoding different locations in the visual field. “Bars,” “blocks,” and “luminosities” allow to decode stimulus sizes on, respectively, 3, 3, and 5 levels. “Outer vs center” is used to study the decoupling of stimulus size and location, trying to distinguish large stimuli in the outer part of the visual field and small stimuli in the central part of the visual field.

### Event related potential (ERP) analysis

ERPs were computed by averaging multiple epochs belonging to the same class, i.e.,

$$Y_{j}^{C,m}[t]=\frac{1}{N_{C}}\sum_{n\;\in \;C}X_{j}^{n,m}\left[t\right],$$

where C indicates the class, m the subject, j the channel, t the time step, and $N_{C}$ the number of samples from class C. A statistical analysis was performed using the MNE function mne.stats.permutation_cluster_test with default parameters to see if there was a significant difference between the responses from two or multiple classes. This analysis was done separately for each subject and recording channel in the occipital and parietal lobes (identified as the channels containing an “O” or a “P” in their identifier).

We chose to show the results relative to subjects 1, 2, and 10 as they yielded the best, worst, and average single-trial decoding accuracies, respectively.

### Single- and multi-trial decoding

Classification was done using a support vector classifier (SVC)^61^ calibrated using the function sklearn.calibration.CalibratedClassifierCV with default parameters (where the calibration happens on the training set).

The spatiotemporal matrix of EEG signals for a trial was reshaped into a one-dimensional array of features, concatenating the time-signal from each EEG channel, considering all channels (not just the occipital and parietal ones). Thus, the EEG signal values corresponding to each couple of time points and channels were treated as an independent feature in the classification.

When the classes used in a decoding task were composed of approximately the same number of stimuli, i.e., the corresponding dataset was balanced, we estimated the decoding accuracy for the given decoding task using a tenfold cross-validation. Balanced decoding tasks are ideally composed of the same number of trials per class, even though on averagefour trials out of the 3000 acquired for each subject were not recorded by the acquisition software, thus resulting in a possible mismatch of a few trials across different classes. Instead, when the dataset corresponding to a given decoding task was heavily unbalanced, the accuracy was estimated using cross validation employing 10 random resamplings (without replacement) of the trials from each class until the number of trials of the least represented class for each decoding task was reached. The resulting number of samples for each decoding task is detailed in supplementary material Table 1.

We standardized the signals in the training and test sets using z-scoring as follows:

$$X_{i,j}^{n,m}\leftarrow \frac{X_{i,j}^{n,m}-\mu_{j}^{m}}{\sigma_{j}^{m}},$$

where we computed the mean and standard deviation for each subject ( m) and channel ( j) on the training set (for standardizing both training and test set) as

$$\mu_{j}^{m}=\frac{1}{N_{i}\cdot N_{n}}\sum_{t}\sum_{n}X_{j}^{n,m}[t],\;\sigma_{j}^{m}=\sqrt{\frac{1}{N_{i}\cdot N_{n}-1}\sum_{t}\sum_{n}{\left(X_{j}^{n,m}[t]-\mu_{j}^{m}\right)}^{2}},$$

where $N_{i}$ is the number of time steps and $N_{n}$ is the number of trials.

In order to determine whether our decoding accuracy was significantly above chance, we compared the decoding accuracy obtained for each task and subject to the accuracy obtained by a random decoder. Such a decoder produced the same number of decoding labels as the given subject for the selected decoding task. We thus compared the ten accuracy values corresponding to the ten different folds/resamplings both for the real subject and for the random decoder using a Wilcoxon test, after checking that each set of ten accuracy values was not Gaussian. The obtained p-values were corrected using Bonferroni correction for the number of subjects, considering each task as an independent scenario.

Additionally, we computed a sensitivity map of our RBF SVMs to the input features, providing a measure of feature importance, using the method introduced in Ref. 62 and employed for EEG decoding in Ref. 63. The computation of such maps was performed using the code from Ref. 63 freely accessible at https://github.com/gretatuckute/DecodingSensitivityMapping/.

In order to compute the predicted labels through multi-trial decoding, for each fold, we considered the test set and predicted the classification probabilities for each sample therein. Then, we combined such probabilities using a sliding window of N trials on the shuffled test set, with N ranging from 2 to 15. Accuracies were calculated based on the combined probabilities using an argmax function, as for single-trial.

The aggregated probability, $p_{k}$, to belong to macro-class k is obtained from the equation

$$p_{k}=\frac{\prod_{i=1}^{N}{\left(\frac{p_{i,k}}{p_{i,M}}\right)}^{1/N}}{\sum_{j=1}^{M}\left[\prod_{i=1}^{N}{\left(\frac{p_{i,j}}{p_{i,M}}\right)}^{1/N}\right]},\;k=1,\;\ldots ,\;M.$$

Here, i is the trial and N the total number of trials to aggregate (here, ranging from 1 to 15). M is the number of alternative macro-classes constituting the given tuning set (e. g., M=2 for coarse left vs right and M=5 for fine left vs right).^41^ The original probabilities, $p_{i,j}$, were obtained after calibrating the trained SVMs through Platt's scaling.^64^ The method used to combine probabilities did not affect calibration.^55^

### Independent component analysis

Even though subjects were instructed to fixate the center of the screen during the whole experiments, residual movement activity might influence decoding scores. To test and ensure that the decoding was based only on the neural correlates of the stimuli, rather than eye movements, we also calculated, for subjects 1, 2, and 10, the decoding accuracy after removing ocular activity (blinks and horizontal eye movements) by means of independent component analysis (ICA).^65^ As ICA algorithm we used the Picard ICA algorithm within the MNE toolbox for Python (function mne.preprocessing.ICA).

## SUPPLEMENTARY MATERIAL

See the supplementary material for further information on experimental design, effect of eye artifact removal on decoding performance, feature importance analyses, and further information on multi-trial performance.

## Data Availability

The data that support the findings of this study are available from the corresponding author upon reasonable request.
